# Gypenosides Protected the Neural Stem Cells in the Subventricular Zone of Neonatal Rats that Were Prenatally Exposed to Ethanol

**DOI:** 10.3390/ijms151221967

**Published:** 2014-11-28

**Authors:** Lun Dong, Kun-Qi Yang, Wen-Yan Fu, Zhen-Hua Shang, Qing-Yu Zhang, Fang-Miao Jing, Lin-Lin Li, Hua Xin, Xiao-Jing Wang

**Affiliations:** 1 Department of Cell Biology, School of Medicine, Shandong University, Jinan 250012, China; E-Mails: 13589030976@163.com (L.D.); kunqiyang@126.com (K.-Q.Y.); fuwenyan411@163.com (W.-Y.F.); shangzhenhua16@126.com (Z.-H.S.); zqy2008512@163.com (Q.-Y.Z.); amber_jfm@163.com (F.-M.J.);13583108986@163.com (L.-L.L.); xinhua@sdu.edu.cn (H.X.); 2 Department of Breast Surgery, Qilu Hospital, Shandong University, Jinan 250012, China

**Keywords:** subventricular zone, cell proliferation, gypenosides, fetal alcohol spectrum disorder

## Abstract

Fetal alcohol spectrum disorder (FASD) can cause severe mental retardation in children who are prenatally exposed to ethanol. The effects of prenatal and early postnatal ethanol exposure on adult hippocampal neurogenesis have been investigated; however, the effects of prenatal ethanol exposure on the subventricular zone (SVZ) have not. Gypenosides (GPs) have been reported to have neuroprotective effects in addition to other bioactivities. The effects of GPs on neural stem cells (NSCs) in the FASD model are unknown. Here, we test the effect of prenatal ethanol exposure on the neonatal SVZ, and the protection potential of GPs on NSCs in FASD rats. Our results show that prenatal ethanol exposure can suppress the cell proliferation and differentiation of neural stem cells in the neonatal SVZ and that GPs (400 mg/kg/day) can significantly increase the cell proliferation and differentiation of neural stem cells inhibited by ethanol. Our data indicate that GPs have neuroprotective effects on the NSCs and can enhance the neurogenesis inhibited by ethanol within the SVZ of neonatal rats. These findings provide new evidence for a potential therapy involving GPs for the treatment of FASD.

## 1. Introduction

The term fetal alcohol spectrum disorder (FASD), first defined in 1973 [1], is used to describe the developmental disorders caused by prenatal exposure to ethanol. The signs of FASD include characteristic facial dysmorphology, prenatal and postnatal growth deficiencies, and central nervous system (CNS) dysfunction [1,2]. FASD is a leading non-genetic cause of mental retardation. Experimental evidence demonstrates that ethanol interferes with many ontogenic phases of brain development, affecting crucial processes, such as neuronal migration, neurogenesis and gliogenesis [3]. Several regions of the developing CNS are known to be affected by ethanol exposure including the cerebral cortex, hippocampus, dorsal root ganglia, and the spinal cord [4].

Neural stem cells (NSCs) are distributed in many areas in the developing brain, such as the cerebral cortex, corpus striatum, hippocampus, olfactory bulb, cerebellum and spinal cord [5]. During the end of the first trimester (gestational days-GDs 1–10 for rodents) through the second trimester (GDs 11–21/22 for mice/rats) of fetal development, NSCs produce the majority of adult neurons. Thus, this period is a specific window of vulnerability to ethanol [6]. An *in vitro* culture system has been used to show that NSCs are the targets of ethanol and to study the effect of ethanol on the developing brain. Human NSCs have proven to be more sensitive to ethanol exposure than astrocytes [7]. For the cultured E13 rat neuroepithelial cells, ethanol blocked the cell proliferation in a dose-dependent manner [8]. Other studies have shown that ethanol alters the cell fate of neural stem/progenitor cells [9,10,11]. One study showed alcohol prevented the normal DNA methylation programming of NSC’s genes and slowed NSC’s differentiation [12]. The dentate gyrus (DG) of the hippocampus and the subventricular zone (SVZ) are two regions in the mammalian brain where neurogenesis occurs throughout life. The effects of prenatal and early postnatal ethanol exposure on adult hippocampal neurogenesis have been investigated [13,14,15,16,17]. Only one study has shown a decrease in cell proliferation in the SVZ in rats that were exposed to ethanol during adolescence [18]. The effects of ethanol exposure during the period of brain development on the SVZ have not been thoroughly investigated.

Gypenosides (GPs) are dammarane-type saponins extracted from Gynostemma pentaphyllum (Thunb) Makino (Cucurbitaceae). The effective component of gypenoside is the hydroxy group at the twentieth or twenty-first carbon in the dammarane-type ring [19]. GPs are widely used in Asia as a component of traditional Chinese medicine. Studies have shown that GPs have antioxidative stress effects in both rat cortical cells treated by glutamate [19] and in the cortex and hippocampal CA1 region in a rat model of chronic cerebral hypoperfusion [20]. Some studies have shown that GPs have neuroprotective effects on dopaminergic neurons in cultures [21] and in the substantia nigra of mouse and rat models of Parkinson’s disease [22,23]. However, whether GPs have a protective function for NSCs in ethanol-treated rat is unknown. In this study, we established the FASD rat model by feeding ethanol by gavage to pregnant rats to investigate the protective function of GPs on NSCs against ethanol-induced toxicity.

## 2. Results and Discussion

### 2.1. Ethanol Treatment Increased the Lethality and Abnormality of the Neonatal Rats

**Figure 1:**
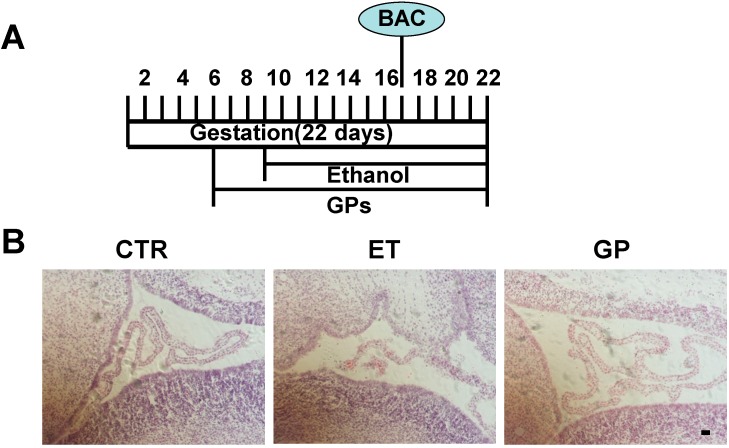
Gypenosides (GPs) protected the cell morphology of the subventricular zone (SVZ) in the neonatal brain of fetal alcohol spectrum disorder (FASD) rats. (**A**) Experimental scheme for the FASD rats. The rats were pre-treated with GPs (400 mg/kg/day) by gavage for G6 prior to treatment by ethanol. Ethanol (50% (*v*/*v*)) was given by oral gavage according 4.0 g/kg body weight from G 9 until birth. The blood alcohol concentrations (BAC) was tested on G17 and (**B**) The representative HE staining for the cell morphology of SVZ. Scale bar = 200 μm.

The rats were treated with GPs from G6 and 50% (*v*/*v*) ethanol from G9 until birth by oral gavage (Figure 1A). At G9, the body weight of the pregnant rats was 300.45 ± 6.45 g for the control group (CTR group), 299.95 ± 6.95 g for the ethanol treatment group (ET group), and 325.56 ± 12.31 g for the GPs treatment group (GP group). There was not a significant difference between these three groups. At G21, the body weight of the pregnant rats was 336.45 ± 14.05 g for the CTR group, 366.55 ± 3.55 g for the ET group, and 370.64 ± 20.78 g for the GP group. Again, there was not a significant difference between these three groups. This suggests that the ethanol treatment had no significant effect on the body weight of the pregnant rats.

The CTR group had a total 38 pups, the ET group produced 55 pups, and the GP group had 57 pups. The lethality rate and abnormality rate were calculated for the P0 pups. In the ET group, the lethality rate was 15.14% ± 8.13% and the abnormality rate was 12.57% ± 5.61%. In the GP group, the lethality rate was 9.70% ± 6.35% and the abnormality rate was 3.66% ± 2.47%. The lethality and abnormality rates in the control group were zero. Although both the lethality and abnormality rates of the GP group were reduced compared to the rates of the ET group, there was no significant difference between these two groups. These data suggest that the ethanol treatment caused the lethality and abnormality in the neonatal rats.

### 2.2. Blood Alcohol Concentration

Tail blood samples were taken 120 min after gavage with ethanol at G17. BAC was determined and calculated by a 7890GC Analyser (gas chromatograph) and reported in mg/dL. The ET group had a peak blood level of 239.45 mg/dL at 120 min, which was well above the legal driving limit of 80 mg/dL. The peak blood level was 215.55 mg/dL in the GP group.

### 2.3. GPs Protected the Cell Morphology of SVZ in the Neonatal Brains Exposed to Ethanol as Embryos

Histological analysis was performed on the P0 brain. H&E staining showed that the lateral ventricular of the ET group was irregular in the coronal sections. The subventricular zone (SVZ) was significantly thin compared to that of the CTR group (Figure 1B). The shape of the lateral ventricular and the thinness of SVZ in the GP group were similar to those in the CTR group (Figure 1B). These data suggest that GPs had protective effects on the cells’ morphology in the SVZ in the neonatal brains exposed to ethanol as embryos.

### 2.4. GPs Increased the Cell Proliferation of Neural Stem Cells in the SVZ, Reversing the Inhibition by Ethanol

**Figure 2:**
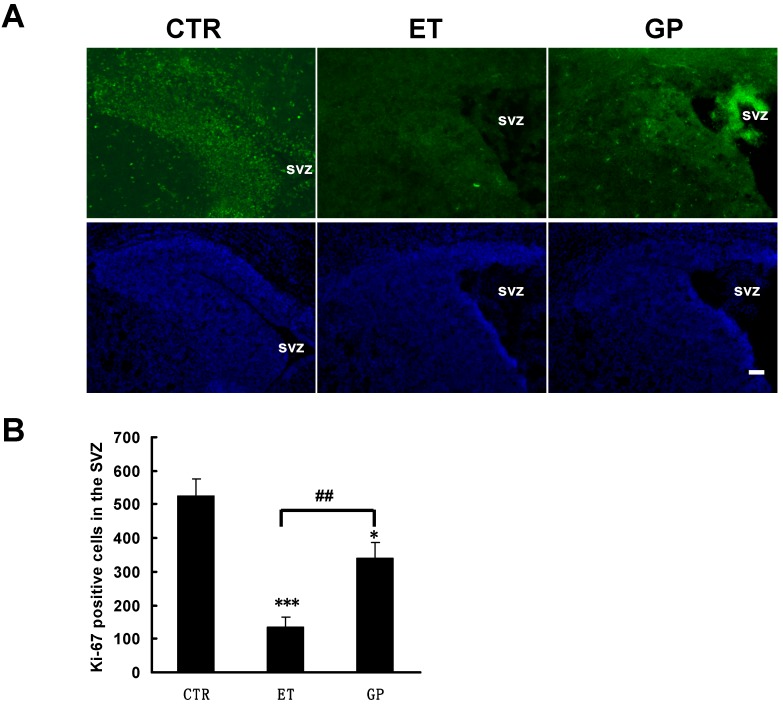
GPs increased the cell proliferation of neural stem cells in the SVZ of neonatal brain that were inhibited by ethanol. (**A**) Immunohistochemical staining for Ki67 was labeled in the SVZ of neonatal FASD rats. DAPI (blue) labeled all nuclei. Scale bar = 200 μm and (**B**) Quantitative analysis of the number of Ki67-positive cells in the SVZ. * *p* < 0.05 and *** *p* < 0.001 *vs.* CTR; ## *p* < 0.01 *vs.* ET group.

The SVZ is one region of neurogenesis in the mammalian brain. During development, neural stem cells proliferate and differentiate into new neurons, and they migrate from the SVZ via the rostral migratory stream (RMS) to the olfactory bulb (OB) and also enter the association necocortex [24,25]. In order to investigate the effects of ethanol on the proliferation of neural stem cells in the SVZ, immunofluorescence staining was performed. Ki-67 is a marker that labels cell proliferation [26]. In the control rats, there were many Ki-67-positive cells in the SVZ. Compared to the CTR group, the ET group and GP group had significantly fewer Ki-67-positive cells in the SVZ (134.83 ± 29.83 *vs*. 523.60 ± 51.98; 339.33 ± 48.31 *vs*. 523.60 ± 51.98; Figure 2A,B). There was a significant difference between the ET group and the GP group (134.83 ± 29.83 *vs*. 339.33 ± 48.31). These data showed that ethanol treatment can reduce cell proliferation in the SVZ of the neonatal brain. Treatment with GPs increased the cell proliferation in the SVZ that is typically inhibited by ethanol. This suggests that GPs have a protective effect on the neural stem cells in the SVZ of the neonatal brain.

### 2.5. GPs Promoted the Cell Differentiation of Neural Stem Cells in the SVZ that Were Inhibited by Ethanol

**Figure 3:**
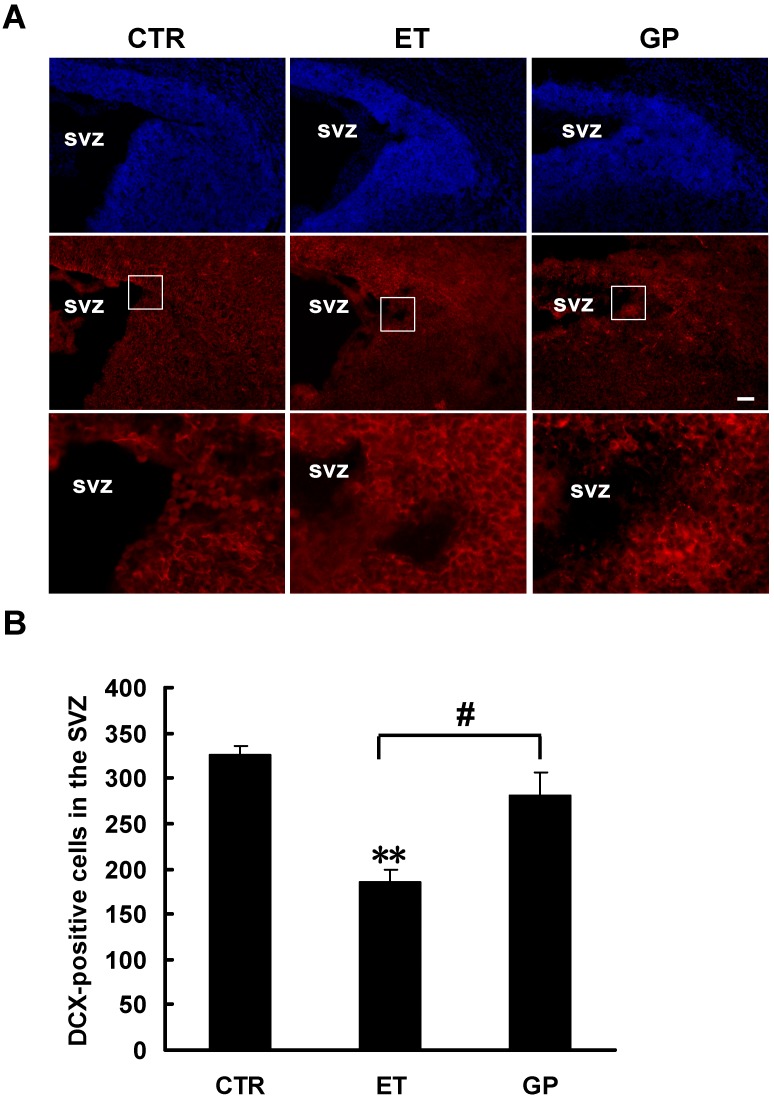
GPs increase the number of DCX-positive cells in the SVZ of the neonatal brain that was inhibited by ethanol. (**A**) Immunohistochemical staining for DCX was labeled in the SVZ of neonatal FASD rats. DAPI (blue) labeled all nuclei. Scale bar = 200 μm. The outlined rectangular fields are respectively shown at higher magnification in the lower pictures; (**B**) Quantitative analysis of the number of DCX-positive cells in the SVZ. ** *p* < 0.01 *vs.* CTR; # *p* < 0.05 *vs.* ET group.

To investigate the effects of ethanol on the cell differentiation in the SVZ, two markers were studied: DCX, a marker of immature newborn neurons [27], and GFAP, a marker of astrocytes [28]. In the control rats, there were many DCX-positive cells in the SVZ of the neonatal brain. Compared to the CTR group, there were significantly fewer DCX-positive cells in the SVZ of the ET group (185.25 ± 12.83 *vs*. 324.95 ± 10.84; Figure 3A,B). The DAPI staining results indicated that the thickness of the SVZ in the ET group was significantly reduced compared to the CTR group. There was not a significant difference in the DCX-positive cells between the CTR group and the GP group (324.95 ± 10.84 *vs*. 280.67 ± 25.86; Figure 3A,B). However, compared to the ET group, the GP group had significantly more DCX-positive cells in the SVZ (185.25 ± 12.83 *vs*. 280.67 ± 25.86; Figure 3A,B).

**Figure 4:**
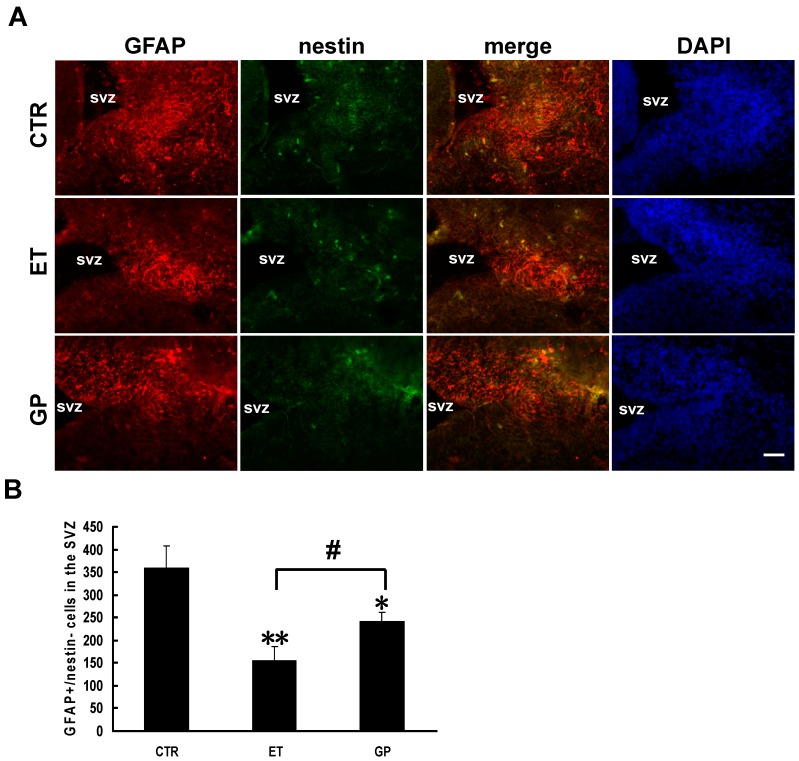
GPs increase the number of GFAP+/nestin- cells in the SVZ of the neonatal brain that was inhibited by ethanol. (**A**) Immunohistochemical double staining for GFAP (red) and nestin (green) was labeled in the SVZ of neonatal FASD rats. DAPI (blue) labeled all nuclei. Scale bar = 50 μm; (**B**) Quantitative analysis of the number of GFAP+/nestin- cells in the SVZ. * *p* < 0.05 and ** *p* < 0.01 *vs.* CTR; # *p* < 0.05 *vs.* ET group.

In the sagittal sections, the GFAP-positive cells were located in the caudal SVZ. Because both NSCs and astrocytes express GFAP, the double-label immunohistochemistry of nestin (the marker of NSCs) and GFAP was performed (Figure 4). The GFAP+/nestin- cells represent astrocytes. There were no GFAP-positive cells in the rostral SVZ. There were more GFAP+/nestin- cells in the CTR group. Compared to the CTR group, there were significantly fewer GFAP+/nestin- cells in the caudal SVZ of both the ET group and the GP group (156.14 ± 30.83 *vs*. 358.17 ± 50.07; 241.75 ± 19.50 *vs*. 358.17 ± 50.07; Figure 4A,B). Compared to the ET group, the GP group had significantly more GFAP+/nestin- cells (241.75 ± 19.50 *vs*. 156.14 ± 30.83; Figure 4A,B). These data show that GPs increased the DCX-positive and GFAP+/nestin- cells in the SVZ of the neonatal brain exposed to ethanol. This suggests that GPs can promote the cell differentiation of neural stem cells in the SVZ that were inhibited by ethanol.

### 2.6. Discussion

In the present study, we evaluated the effects of prenatal ethanol exposure on the neural stem cells in the neonatal SVZ and the protective effects of GPs (400 mg/kg/day) on FASD rats. The present data provide the first evidence that prenatal ethanol exposure can suppress the cell proliferation and differentiation of neural stem cells in the neonatal SVZ and that GPs (400 mg/kg/day) can significantly increase the cell proliferation and differentiation of neural stem cells that were inhibited by ethanol.

Exposure to ethanol during pregnancy can be devastating to the developing nervous system, leading to significant central nervous system dysfunction. Compared to effects on the hippocampus, the effects of ethanol exposure during development on the second neurogenic zone SVZ have not been thoroughly investigated. Prolonged treatment (7 weeks) with ethanol in adult rats can induce the long-term reduction of the SVZ stem cell pool and suppress forebrain neurogenesis [29]. When treated with acute ethanol during adolescence, rats have reduced NSC proliferation in the SVZ and hippocampus [18]. These data showed that the SVZ region is sensitive to the effect of ethanol. In this study, we found that prenatal ethanol exposure can suppress the cell proliferation and differentiation of neural stem cells in the neonatal SVZ. In previous published work, prenatal ethanol exposure has been shown to decrease the thickness and cell proliferation rate within the ventricular zone (VZ) and to decrease proliferation within the SVZ [30,31]. The difference between these data may relate to two factors: the peak BAC and the definition of the SVZ. The peak BAC depends on both the dose and pattern of ethanol exposure. Different doses of ethanol can have different effects on the cell proliferation and differentiation in the SVZ. The SVZ is a cytoarchitectonically defined region in the brain that is situated adjacent to the lateral ventricles. Over the past decades, the cells of the SVZ have been intensely studied as a consequence of the discovery that the SVZ harbors NSCs [32]. Many studies describe this region using different terms, and the boundary between the VZ and SVZ is not easy to identify under the light microscope. Thus, some papers include the VZ cells in the SVZ [33].

Our results suggest that GPs have protective potential for the NSCs in the neonatal SVZ of FASD rats. GPs (400 mg/kg/day) can significantly increase neural stem cell proliferation and differentiation in the neonatal SVZ that has been inhibited by ethanol. In the stroke model, we also found that GPs (400 mg/kg/day) can significantly enhance neurogenesis in the SVZ in MCAO adult rats [34]. The mechanism of GPs on the NSCs remains elusive. In vitro experiments, GPs was found having the effect of antioxidative stress not only in the glutamate-induced apoptosis of primary cortical cells but also in the MPP+-induced injury of dopaminergic neurons [19,20]. In a PD mouse model, GPs was also found to increase antioxidation in the substantia nigra [23]. Studies showed that GPs had the effects of antioxidative stress and antiastrocytic activation in the chronic cerebral hypoperfusion rats [20,35]. Besides these effects, other multiple mechanisms may be involved in the protective properties of GPs. Whether GPs operates through the similar effects on NSCs in the SVZ is unclear. The precise mechanism underlying the neuroprotective effects of GPs on NSCs requires further investigation.

In conclusion, this study is the first to reveal that GPs have protective potential for the NSCs in the neonatal SVZ of FASD rats. Prenatal ethanol exposure can suppress the cell proliferation and differentiation of NSCs in the neonatal SVZ. GPs (400 mg/kg/day) can significantly increase the cell proliferation and differentiation of NSCs inhibited by ethanol. Further studies exploring the mechanisms underlying the effects of GPs on NSCs may lead to a better understanding of the potential for therapies using GPs in the treatment of FASD.

## 3. Experimental Section

### 3.1. Experimental Materials

Pregnant Wistar rats were obtained from the Experimental Animal Center of Shandong University (Jinan, China). The rats were kept under standard lighting conditions (12 h light/dark cycles). This study was approved by the ethics committee of the School of Medicine of Shandong University. All procedures were approved by the Institutional Animal Care and Use Committee (IACUC) of Shandong University. GPs (colorless powder, purity 98%) were purchased from JIAHE PHYTOCHEM (Shanxi, China). GPs dissolved in distilled water and boiled to concentrate till 100 mg/mL and then stored in −20 °C.

### 3.2. Study Design for Animal Models

The rats were randomly divided into three groups: a CTR group (*n* = 5), an ET group (*n* = 6) and a GP group (*n* = 6). The rats in the ET group were fed 50% (*v*/*v*) ethanol at a ratio of 4.0 g/kg body weight from gestational day G9 until birth by oral gavage. The GP group rats were fed GPs at a ratio of 400 mg/kg body weight from G6. The dose of GPs was applied according to other reports [34,35]. After G9, the rats were treated with ethanol, just as the ET group was, two hours after the GPs feeding. The CTR group was treated with distilled water. Rats were weighed every day, and maternal BAC were determined on G17.

### 3.3. H&E Staining

The day of birth for any given litter was designated as postnatal day (P) 0 for those pups. The pups were perfused with 4% paraformaldehyde (PFA), and their brains were dissected, followed by overnight postfixation in 4% PFA at 4 °C. The brains were cryoprotected in 20% sucrose, and serial sections were prepared at 16 µm for cryostat sections. Every tenth slide was stained by H&E. Adjacent sections were subjected to immunohistochemistry analysis. For the H&E staining, the slides were treated sequentially with 100% ethanol, 95% ethanol, distilled water, hematoxylin, clarifier solution, bluing solution, eosin, 95% ethanol, 100% ethanol and xylene. Finally the coverslips were mounted on the slides with mounting medium.

### 3.4. Immunohistochemistry

Serial 16 μm coronal sections were cut with cryostat and stored at −80 °C. For the immunohistochemistry staining, the slides were blocked with PBS containing 0.3% Triton X-100 and 3% fetal blood serum for 1 h. The sections were then incubated with the mouse monoclonal antibody against human Ki-67 (1:100, BD Pharmingen™, Franklin Lakes, NJ, USA), the rabbit monoclonal anti-doublecortin (DCX, 1:500; Cell Signaling Technology, Beverly, MA, USA), the mouse monoclonal anti-nestin (1:500; Millipore, Billerica, MA, USA) and the rabbit polyclonal anti-glial fibrillary acidic protein (GFAP, 1:1000; Millipore, Billerica, MA, USA) at 4 °C overnight and were washed three times with PBS. Next, the slides were incubated with Alexa Fluor 488-conjugated goat anti-mouse IgG (H + L) (1:1000; Invitrogen, Grand Island, NY, USA) and Alexa Fluor 594-conjugated goat anti-rabbit IgG (H + L) (1:1000; Invitrogen, Grand Island, NY, USA) for 2 h at room temperature. Sections were mounted with Vectashield (Vector, Burlingame, CA, USA), and 4',6-diamidino-2-phenylindole (DAPI) was used to counterstain the nuclei.

### 3.5. Microscopy Analysis

Coronal or sagittal sections were serially cut at 16 µm thickness to cover the whole SVZ. One slide had adhered three sections. One rat pup had about thirty-forty slides to cover the whole SVZ. Every tenth slide was collected for staining. HE staining was used to choose the similar SVZ structure across mice. Three sets of slides from each rat were separately used for Ki-67, DCX and GFAP/nestin staining. To quantify the Ki-67, DCX and GFAP/nestin labeling, images were digitally captured using a Nikon 80i light microscope equipped with a CCD camera (Photometrics, Tucson, AZ, USA). The images were imported into NIH Image J for blinded cell counting. For cell counting, 100× magnification was used. The SVZ areas were defined by DAPI staining. In coronal section the NSCs mainly located in dorsolateral SVZ and striatal SVZ [32,36]. So the areas of dorsolateral SVZ and striatal SVZ were used for counting the Ki-67- and DCX-positive cells. In the sagittal section the anterior SVZ generates the migrating olfactory bulb neuronal precursors, and the caudal SVZ generates glial cells [37,38]. The areas of caudal SVZ was used for counting the GFAP+/nestin- cells. The total cell number of Ki-67+, DCX+ and GFAP+/nestin- cells in three or four slides per rat was counted in the SVZ for each group (*n* = 3).

### 3.6. Statistical Analyses

All values in the text and figures represent the mean ± SEM. Data were analyzed with unpaired Student’s *t*-test or one-way ANOVA. Data analyses were performed using SPSS software. Statistical significance was defined at *p* < 0.05.

## 4. Conclusions

Prenatal ethanol exposure can suppress the cell proliferation and differentiation of NSCs in the neonatal SVZ. GPs have protective potential for the NSCs in the neonatal SVZ of FASD rats. These findings suggest that GPs have potential application in FASD therapeutics.
